# Current and Potential Applications of Monoterpenes and Their Derivatives in Oral Health Care

**DOI:** 10.3390/molecules28207178

**Published:** 2023-10-19

**Authors:** Wiktoria Potocka, Zainab Assy, Floris J. Bikker, Marja L. Laine

**Affiliations:** 1 Department of Oral Biochemistry, Academic Centre for Dentistry Amsterdam, University of Amsterdam and VU University Amsterdam, Gustav Mahlerlaan 3004, 1081 LA Amsterdam, The Netherlands; z.assy@acta.nl (Z.A.); f.bikker@acta.nl (F.J.B.); 2 Department of Periodontology, Academic Centre for Dentistry Amsterdam, University of Amsterdam and VU University Amsterdam, Gustav Mahlerlaan 3004, 1081 LA Amsterdam, The Netherlands; m.laine@acta.nl

**Keywords:** oral health, dentistry, terpene, monoterpene, monoterpenoid, plant product, plant oil, volatile oil

## Abstract

Plant products have been employed in medicine for centuries. As the world becomes more health-conscious, there is a growing interest in natural and minimally processed products for oral health care. This has led to an increase in research into the bioactive compounds found in plant products, particularly monoterpenes. Monoterpenes are known to have beneficial biological properties, but the specific mechanisms by which they exert their effects are not yet fully understood. Despite this, some monoterpenes are already being used in oral health care. For example, thymol, which has antibacterial properties, is an ingredient in varnish used for caries prevention. In addition to this, monoterpenes have also demonstrated antifungal, antiviral, and anti-inflammatory properties, making them versatile for various applications. As research continues, there is potential for even more discoveries regarding the benefits of monoterpenes in oral health care. This narrative literature review gives an overview of the biological properties and current and potential applications of selected monoterpenes and their derivatives in oral health care. These compounds demonstrate promising potential for future medical development, and their applications in future research are expected to expand.

## 1. Introduction

Plants, as well as their derived compounds have been utilized in medicine and dentistry for centuries. For example, chew sticks, such as miswak, are thought to have been used in Babylonia as early as 3500 B.C. [1]. These sticks were made from twigs typically derived from aromatic trees such as the toothbrush tree (*Salvadora persica*) and applied as a toothbrush-like tool to mechanically remove (inter)dental debris. In addition, during brushing and chewing, the oils of the chew sticks were applied to the tooth surface and released into the saliva, potentially providing antimicrobial benefits and supporting oral health [2,3]. In turn, clove (*Syzygium aromaticum*) has been used as a natural anesthetic for treating toothache [4]. In addition to its analgesic properties, clove oil was found to possess anti-inflammatory and antioxidant properties and to aid in the wound-healing process [5,6]. In line with this, the practice of using clove oil to treat dental caries has been common since the sixteenth century, and in 1834, eugenol, which is the main active ingredient of clove oil, was extracted [4]. Eugenol was later established to belong to the class of terpenes [7].

Terpenes are a large and diverse group of naturally occurring compounds, generally found in plants. Over 30,000 terpenes have been described in the literature so far [8]. They are also termed isoprenoids, due to their structure comprising 2-methylbutane residues, often referred to as isoprene units, ((C_5_)_n_) [8]. Monoterpenes, belonging to a class of terpenes, consist of two linked isoprene units, which can be cyclized and oxidized in several ways. Monoterpenes and their derivatives can be found in plants like true lavender (*Lavandula angustifolia*) or basil (*Ocimum basilicum*). Depending on their structure, they are classified as acyclic, monocyclic, or bicyclic [7,9,10]. With over 1500 monoterpenes documented, their diverse biological and therapeutic properties make them promising candidates for use in medicine and dentistry [11].

The demand for natural and organic products has seen a steady rise, with the global market value for natural cosmetics and personal care items expected to grow by 40% between 2021 and 2027 [12,13]. This growing trend also extends to oral care products [14]. Consumers are showing a keen interest in products such as turmeric toothpaste [15,16] and oil-pulling blends enriched with volatile oils [17]. In line with this, interest in plant- and, in particular, monoterpene-based products is emerging, which often stems from the traditional use of home remedies that have been passed down through generations. Despite the recent surge in interest, many of these monoterpenes remain relatively unexplored in terms of their potential benefits and applications. Overall, the global market’s shift towards natural and organic options, particularly in the cosmetics, personal care, and oral care sectors, signifies a growing preference for products that are perceived as healthier and more environmentally friendly. This trend also presents exciting opportunities for the further exploration and development of novel monoterpene-based products.

In this review, the biological properties, practical uses, and applications of monoterpenes in the context of oral health care will be explored. These findings will provide an overview of the current literature and reveal the potential benefits of using monoterpenes in oral health care settings.

## 2. Biological Properties

The extraction of monoterpenes and their derivatives (monoterpenoids) from plant products has led to the discovery of their wide range of biological properties, some of which can be seen in Table 1. In this review, we classified these molecules based on their biological activity: antimicrobial and antiseptic, anti-cancer, anti-inflammatory, and analgesic and antinociceptive activities.

### 2.1. Antimicrobial and Antiseptic Activity

Numerous studies have demonstrated the antimicrobial potency of monoterpenes and monoterpenoids. Monoterpenoids are modified monoterpenes containing different functional groups such as alcohols, carboxylic acids, ketones, aldehydes, and phenols. Some of these compounds exhibit a broad-spectrum antimicrobial effect [96,97]. With an increasing prevalence of multi-drug-resistant pathogens, these compounds have drawn attention as alternative therapeutic agents. Taking into consideration that over 700 species of bacteria occupy the oral cavity [98], their eubiosis is imperative for the host’s oral health. Microbial dysbiosis has been involved in a broad range of oral diseases including caries, periodontal disease, and halitosis but also conditions such as odontogenic maxillary sinusitis, dentoalveolar abscesses, periradicular pathosis, jaw osteonecrosis, and jaw osteomyelitis [23]. Even systemic diseases, such as Alzheimer’s, cancer, cardiovascular disease, and diabetes, have been linked to oral microbial dysbiosis [99,100].

#### 2.1.1. Antibacterial Activity

For centuries, plant-origin compounds have been employed in the treatment of oral bacterial infections [4,86,101]. For example, a screening of medicinal plants occurring on the Isle of Arran in Scotland, based on Meddygion Myddvai (a 14th-century Welsh manuscript used to treat conditions related to microbial infections), found that Juniper berries (*Juniperus communis* L.) exhibited antibacterial activity against *Staphylococcus aureus* and *Escherichia coli* [86]. The antibacterial properties of this plant were attributed to the activity of sabinene, which is a natural bicyclic monoterpene found in juniper berries. To investigate the antibacterial properties of sabinene, a research group in Korea assessed the compound in vitro against the cariogenic *Streptococcus mutans* [89]. High concentrations of sabinene inhibited bacterial growth and biofilm formation and caused the downregulation of genes necessary for biofilm formation [89]. Another in vitro study investigated the synergistic effect of eugenol applied in combination with either ampicillin or gentamicin against the *Streptococcus* species *S. mutans*, *Streptococcus sanguinis*, *Streptococcus sobrinus*, *Streptococcus ratti*, *Streptococcus criceti*, *Streptococcus anginosus*, *Streptococcus gordonii* and the periodontitis-associated bacterial species *Aggregatibacter actinomycetemcomitans*, *Fusobacterium nucleatum*, *Prevotella intermedia*, and *Porphyromonas gingivalis* [55]. It was found that eugenol exhibited bactericidal and bacteriostatic activity. In like manner, hinokitiol (β-thujaplicin), a volatile oil component from a tropolone-derived monoterpenoid found naturally in cypress plants, inhibited the growth of oral bacteria, such as *S. mutans*, *S. sobrinus*, *P. gingivalis*, *A. actinomycetemcomitans*, *F. nucleatum*, and *P. intermedia* in vitro [64]. Furthermore, hinokitiol had a bactericidal effect against *S. mutans*, *S. sobrinus*, *A. actinomycetemcomitans*, *F. nucleatum*, and *P. intermedia*. Monoterpenoid thymol was also found to have antibacterial potential against *S. mutans* both in a single infection model and in co-infection with *Candida albicans* in vitro and in vivo [92].

#### 2.1.2. Antifungal Activity

The prevalence of invasive fungal infections is estimated to be 1 billion annually, resulting in 1.5 million deaths worldwide [102,103]. The opportunistic pathogenic yeast *C. albicans*, responsible for causing candidiasis in the mouth, throat, and esophagus, has recently been included in the World Health Organization (WHO) fungal priority pathogen list due to its high antifungal resistance and the number of deaths caused [103]. Patients with dry mouth symptoms are particularly susceptible to oral candidiasis due to reduced salivary secretion [104]. Given the severity of the problem, novel therapies are being sought to control the infection spread and drug resistance.

The results of a screening of the antifungal properties of 16 monoterpenes against *Candida* species, including *C. albicans*, *Candida dubliniensis*, *Candida glabrata*, *Candida guilliermondii*, *Candida krusei*, and *Candida tropicalis*, revealed that (±)-limonene, (+)-α-pinene, and (±)-citronellol were the most effective inhibitors of fungal growth [24]. The inhibition of the growth of *Candida auris* was tested by combining phenolic monoterpenes with antifungal agents [95,105]. Carvacrol, found in oils from oregano (*Origanum vulgare*) and thyme (*Thymus vulgaris*), decreased the surface adherence levels and proteinase production of *C. auris* [105]. Furthermore, carvacrol exhibited either synergistic or additive activity when used together with antifungals such as fluconazole, amphotericin B, nystatin, and caspofungin. Thymol was also found to have anti-candidal activity. In vitro, thymol reduced the ability of *Candida tropicalis* and *C. albicans* to form biofilms, resulting in the disaggregation and deformation of fungal cells and decreased hyphae formation [95]. Furthermore, the presence of thymol reduced the integrity of pre-formed biofilms of both *Candida* species. In combination with the antifungal drug fluconazole, thymol had a synergistic effect on the biofilm and planktonic modes of growth of *C. albicans* and *C. tropicalis*. In turn, the monoterpene hinokitiol exhibited inhibitory activity against fluconazole-sensitive and fluconazole-resistant *Candida* species by impeding the fungal respiration and chelating the fungal intracellular iron in vitro [66]. As iron homeostasis is crucial for fungal survival, its perturbation is an efficient strategy for antifungal treatment [106]. Additionally, hinokitiol lowered the fungal burden in the *Galleria mellonella* larvae model infected with a *C. albicans* strain, compared to control and fluconazole treatment [66]. Further investigation revealed that hinokitiol chelated intracellular iron in *C. albicans* and inhibited mitochondrial respiration, by which the fungal growth was impeded.

#### 2.1.3. Antiviral Activity

Viral infections of the oral mucosa are often present as blistering or ulceration. For example, herpes simplex virus type 1 (HSV-1) is a common infection transmitted through oral-to-oral contact, causing oral herpes in and around the mouth. Other viral infections that can cause blistering or ulceration of the oral mucosa include those caused by the varicella-zoster virus, coxsackievirus, and cytomegalovirus [107].

In a therapeutic screening of selected monoterpenes against HSV-1 in vitro, 1 h incubation of HSV-1 with monoterpenes resulted in 80–90% reduction of viral plaque when treated with α-terpinene, γ-terpinene, α-pinene, p-cymene, terpinen-4-ol, α-terpineol, thymol, citral, or eucalyptol (1,8-cineole) [26]. However, in an assay using the host African green monkey kidney RC-37 cell line treated with the above-mentioned monoterpenes prior to HSV-1 infection, only α-pinene caused a reduction in viral plaque by approximately 35%. α-Pinene likely killed the virus either by interfering with the virion envelope or by masking necessary viral structures [26]. In another experiment designed to impede viral replication cycles, only the presence of either α-pinene or eucalyptol reduced the viral plaque [26]. Further screenings against HSV-1 demonstrated that β-pinene and limonene had inhibitory potential against HSV-1 in RC-37 cells in vitro, possibly via interference with the virion envelope or by masking necessary viral structures [35]. On the other hand, isoborneol, a stereoisomer of borneol and eucalyptol, exhibited virucidal activity against HSV-1 in an in vitro assay. Isoborneol inhibited viral replication through the glycosylation of viral polypeptides of HSV-1 in Vero cells (African green monkey kidney epithelial cells) [69]. Furthermore, the Greek sage (*Salvia fruticosa*) components eucalyptol, camphor, and thujone (α- and β-thujone diastereomers mixture) exhibited viral inhibition when applied to HSV-1-infected Vero cells, with thujone having the highest activity [30]. Two monoterpenoids, perillyl alcohol and perillic acid, were also found to exhibit anti-HSV-1 potential by inhibiting the release of infective virion particles from Vero cells [82].

Another group of viruses that can cause oral ulcerations is that of the coxsackieviruses [107]. Some strains of coxsackievirus A and B can cause herpangina, characterized by aphthous-like ulcerations in the soft palate. Menthol was found to inhibit the coxsackievirus B3 in HeLa cells (epithelial carcinoma) and C57BL/6 mice [76]. Menthol showed antiviral effects by impairing mitochondrial fission in vitro and by decreasing the pancreatic viral titers measured by plaque assays in pancreatic homogenates in vivo. Furthermore, two enantiomers of limonene, R-(+)-limonene and S-(−)-limonene, showed antiviral potency against the coxsackievirus B3 and B4 in HEp-2 (epithelial carcinoma) cells [74].

### 2.2. Anti-Cancer Activity

Over 377,700 cases of lip and oral cavity cancers were reported in 2020 globally, making them the 16th most common malignancies [108]. In the United States in 2022, the five-year survival rate following the diagnosis of oral cavity or pharynx cancer was 67% [109]. Surgery and radiotherapy are the primary treatment modalities for oral cancers; however, these interventions are invasive and affect the daily functioning of the individual treated. Therefore, less-invasive pharmacological therapies are being explored.

In a study assessing the anti-cancer potential of compounds against oral cancer cell lines, hinokitiol was observed to be cytotoxic towards HS3, SAS, and SCC4 human tongue squamous carcinoma cell lines, while exhibiting lower cytotoxicity towards normal human oral keratinocytes [62]. On the other hand, (−)-β-pinene displayed comparable levels of cytotoxicity upon application to SCC9 and SCC25 human tongue squamous carcinoma cells and primary human fibroblasts [34]. Treatment with (−)-β-pinene resulted in increased cytotoxicity against cancerous cells when compared to dimethyl sulfoxide (DMSO) control treatment and treatments with caspase inhibitors. Furthermore, SCC9 cells had a significantly higher number of pyknotic nuclei than the control.

The effects of thymol on cell proliferation were evaluated in vitro using Cal27, SCC4, and SCC9 oral squamous cell carcinoma lines, where thymol was able to significantly decrease cell proliferation by mitochondria-mediated apoptosis [94]. On the other hand, an in vivo experiment in mice with Cal27 tumor xenografts demonstrated that thymol decreased tumor volume, including both cancerous as well as normal cells, with a significant reduction after 16 days. Additionally, thymol increased the number of apoptotic tumor cells when compared to the control. Another terpene, acyclic monoterpene geraniol, reduced the protein levels of molecules associated with tumors, such as p65 subunit nuclear factor kappa-light-chain-enhancer of activated B cells (NF-κB-p65) in the nucleus, tumor necrosis factor-α (TNF-α), interleukin-1β (IL-1β), cyclooxygenase-2 (COX-2) and inducible nitric oxide synthase (iNOS/NOS2) in a Wistar rat model of 4-nitroquinoline-1-oxide (4NQO)-induced tongue carcinogenesis when compared to the control [59]. As the upregulation of the nuclear factor kappa-light-chain-enhancer of activated B cells (NF-κB) pathway was associated with tumor promotion, the ability of geraniol to decrease the NF-κB-p65 levels in the nucleus would suggest its potential anti-tumor capacity.

### 2.3. Anti-Inflammatory Activity

Anti-inflammatory drugs are essential for mitigating inflammation and oxidative stress in tissues. Nonsteroidal anti-inflammatory drugs are frequently utilized in dentistry for pain relief; however, they can induce some negative side effects, including gastrointestinal problems, kidney disease, headaches, dizziness, and cardiovascular problems [110]. Because of the potential side effects, new compounds are being studied to substitute the commonly employed anti-inflammatory drugs.

Sage (*Salvia officinalis* L.) extracts have been traditionally employed as home remedies for the relief of inflammation and discomfort associated with gingivitis [31]. Evidence suggests that sage possesses analgesic and anti-inflammatory properties. Sage is rich in monoterpenes such as eucalyptol, borneol, camphor, and α-/β-thujone. The in vitro application of these monoterpenes to human gingival fibroblasts reduced interleukin-6 (IL-6) receptor and interleukin-8 (IL-8) receptor release. Elevated levels of IL-6 and IL-8 are often associated with periodontitis [111,112,113]. α-Terpineol has a characteristic smell reminiscent of lilac and can be isolated from pine and bitter orange oils, as well as lime fruits, in small quantities. An in vitro study using a carcinoma buccal (KB) cell line reported a reduction in the intracellular production of IL-6 in KB cells treated with α-terpineol when compared to the control [29]. Furthermore, it was found to decrease the gene expression of the IL-6 receptor.

### 2.4. Analgesic and Antinociceptive Activity

The term orofacial pain encompasses any pain experienced in the mouth, jaws, or face and is frequently caused by dental conditions. Despite recent advancements in research, the management of dental-related pain remains challenging and can significantly impact an individual’s quality of life [114]. The effective treatment of orofacial pain requires a comprehensive understanding of the underlying causes and an accurate diagnosis.

An in vivo study evaluated the analgesic effects of a β-cyclodextrin-containing carvacrol complex (CARV-βCD) in a Swiss mouse model of induced orofacial nociception [43]. Nociception is often assessed by measuring either the number of rubbings or the time spent by the animals rubbing the injected area with their fore or hind paws [115]. It is assessed over the period of two phases, the first phase lasting most commonly from 0 to 5 min, and the second phase from 15 to 40 min after the injection. The research found that CARV-βCD significantly reduced the number of nociceptive behaviors in Swiss mice with formalin-induced nociception, whereas carvacrol alone did not [43]. Conversely, in cases of capsaicin- and glutamate-induced orofacial nociception, both CARV-βCD and carvacrol significantly decreased the number of face rubbings. The mode of action of the CARV-βCD complex is thought to be via opioid, vanilloid, or glutamate systems [43]. Another study assessed the ability of citronellal to alleviate pain induced by formaldehyde, capsaicin, and glutamate in Swiss mice [47]. Citronellal was found to reduce the number of face rubbings due to pain caused by all three agents, which are thought to either inhibit substance P release or directly block action of neurokinin-1 (NK-1) receptor [47]. In vivo research in Wister rats assessed the effects of the administration of α-pinene alone and in combination with either bicuculline or naloxone on capsaicin-induced dental pulp nociception. It was found that treatment with α-pinene alone decreased the cumulative time of nociceptive behaviors. The mechanism of modulation may to be related to interactions with γ-aminobutyric acid type A (GABA_A_) receptors [23]. On the other hand, myrtenol was only able to decrease the face rubbing time in the second phase of the formalin test [79]. Myrtenol is thought to reduce orofacial nociception and inflammation through cytokine inhibition and the p38 mitogen-activated protein kinase (p38-MAPK) signal transduction pathway in trigeminal ganglia. Myrtenol also decreased IL-1β and myeloperoxidase (MPO) activity, both being inflammatory indicators.

## 3. Use in Dentistry and Oral Health Care

**Figure 1:**
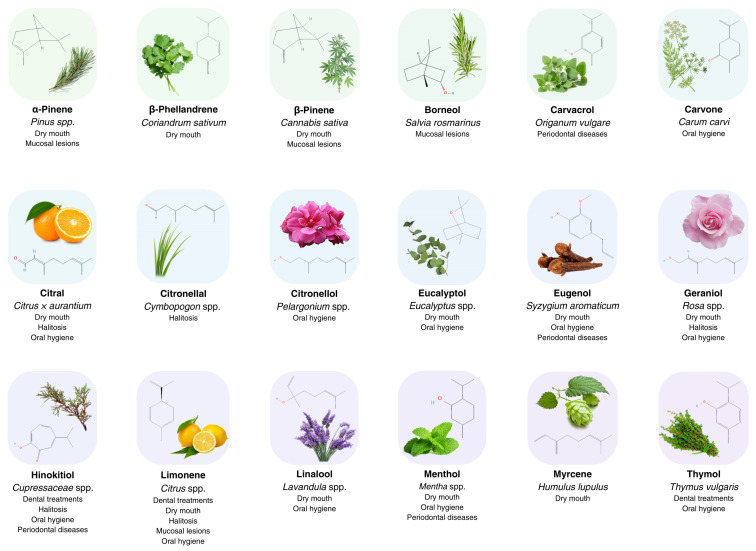
Application of selected monoterpenes and monoterpenoids in oral health care and dentistry. Chemical structures were obtained from PubChem [18], and images from PNGegg [123].

Natural products, including monoterpenes, can be found in both professional and over-the-counter consumer products within all fields of oral health care (Figure 1). In this review, their applications were classified based on their utilization, such as oral hygiene, halitosis, dental treatments, periodontal disease, mucosal lesions, and dry mouth.

### 3.1. Oral Hygiene

Monoterpenes, monoterpenoids, and volatile oils are commonly used as flavorings and perfuming agents in oral health care products. To exemplify, menthol, citral, limonene, hinokitiol, and eugenol, among others, can often be found in toothpaste, mouthwashes, and oral gels. Although there are insufficient appropriate data to substantiate the health benefits of herbal components in oral health care products [116], monoterpenes are commonly added to everyday care products in the form of volatile oils. It is worth noting that many manufacturers do not report the specific compounds present in oral care products. For example, toothpaste can contain mint oil; yet, menthol, the main ingredient, would not be reported. Despite that, it is safe to assume that monoterpenes and monoterpenoids can be found in a wide range of oral care products as fragrances and flavorings.

Toothpaste frequently contains menthol or volatile oils rich in menthol, acting as flavors and fragrances. Other monoterpenes and monoterpenoids such as carvone (0.00005–0.35%) [117] and hinokitiol (0.01–0.20%) [118] can also be found in toothpaste ingredients acting as antibacterial agents. On the other hand, limonene, linalool, citral, geraniol, and citronellol are employed as fragrance ingredients [14]. Toothpaste tablets, which are an alternative to traditional toothpaste, contain low concentrations of monoterpenes and monoterpenoids including menthol, linalool, citral, citronellol, limonene, and geraniol [119,120]. Both toothpaste and toothpaste tablets often contain volatile oils like southern blue gum leaf (*Eucalyptus globulus*), peppermint (*Mentha × piperita*), and lemon (*Citrus limon*), which are abundant in monoterpenes and their derivatives, i.e., respectively, eucalyptol, menthol, and limonene [14]. Menthol and “fresh”-smelling monoterpenes may also be infused into the bristles of toothbrushes and interdental brushes.

Mouthwashes are a staple product line in oral care, with Listerine (McNeil Consumer Healthcare, United States) being one of the most well-known brands. It contains monoterpenes and monoterpenoids such as eucalyptol, menthol, and thymol [121]. Other brands also use carvone, citral, citronellol, eugenol, geraniol, limonene, and linalool [122]. Additionally, monoterpene- and monoterpenoid-rich volatile oils are used, including clove (*S. aromaticum*), rosemary (*Salvia rosmarinus*), common sage (*S. officinalis*), Spanish sage (*Salvia lavandulaefolia*), and common thyme (*T. vulgaris*).

### 3.2. Halitosis

Intra-oral halitosis, bad breath, is often a symptom of bad oral hygiene and/or health issues within the oral cavity and can negatively impact an individual’s social functioning and quality of life. It is estimated that around 30% of the population suffers from halitosis, ranging from mild to severe [124]. Treatment involves addressing the underlying causes, including oral hygiene, periodontal diseases, endodontic infections, and dry mouth [125].

The efficacy of an antibacterial hinokitiol-containing gel on the severity of oral malodor was assessed in patients with diagnosed intra-oral halitosis using an organoleptic test (OLT) and gas chromatography [118]. The treatment group using hinokitiol had significantly lower OLT scores than the control group. This finding was confirmed using gas chromatography; the levels of hydrogen sulfide and methyl mercaptan, which are compounds associated with intra-oral halitosis, were decreased after the hinokitiol treatment. Additionally, in the same study, the use of hinokitiol reduced the number of sites with bleeding on probing (BOP), the average probing pocket depth, and the plaque index after 28 days. Thymoquinone, a monoterpenic quinone found in black cumin oil, was shown to have deodorizing activity and to be able to reduce the smell of methyl mercaptan by 80% [126].

Citrus-like scents, such as those found in citronellal, limonene, or citral, were identified as potential candidates for masking malodor [127]. For this purpose, odor sensor mice were trained to distinguish the smell of dimethyl sulfide, a volatile sulfur compound involved in bad breath. The mice were unable to distinguish the smell of dimethyl sulfide when citronellal, limonene, or citral was applied to the malodorous sample. The potency of lemon oil, which contains limonene as its main constituent [71], was evaluated in saliva from patients with bad breath [128]. The lemon volatile oil was found to reduce the levels of volatile sulfur compounds and bacterial growth and inhibit biofilm formation. Finally, though a lemongrass oil mouth rinse was found to significantly decrease volatile sulfur compounds over time, it did not exhibit antimicrobial potential against halitosis-associated microorganisms despite containing high levels of the monoterpenoids α-citral, β-citral, and geraniol [129].

### 3.3. Dental Treatments

Caries is a widespread condition affecting most of the worldwide population. Over 2 billion people are affected by untreated dental caries in permanent dentition, making it the most prevalent condition in the world [130]. Severe untreated cases of early childhood caries (ECC) and the consequent inflammatory reactions from pulp infections are contributing factors to stunting and underweight in children [130].

The monoterpenoid thymol is currently being considered as a potential therapeutic for the management of ECC [92] due to its antibacterial properties [92,93]. In an in vitro analysis, thymol inhibited the growth of and killed the cariogenic *S. mutans* and opportunistic *C. albicans*. It is thought that there might be an association between ECC and *S. mutans* and *C. albicans* colonization [131,132,133,134]. In this study, thymol interrupted biofilm formation in the presence of human saliva, suppressed the transition of *C. albicans* cells to a hyphal form, and decreased the acid production in *S. mutans* [92]. Furthermore, genes associated with the virulence of these pathogens were downregulated. The application of a chlorhexidine–thymol-containing varnish in carious lesions was also analyzed in children [135,136,137]. Both thymol and chlorhexidine have antimicrobial effects [138]. A chlorhexidine–thymol varnish, Cervitec (Ivoclar, Liechtenstein), was more effective than a fluoride varnish in decreasing the levels of *S. mutans* [135,137]. However, due to high standard deviations, there was no significant difference in the occurrence of caries between the test and the control groups [135,136,137]. Comparable studies were carried out in elderly patients [139,140]. The application of the chlorhexidine–thymol varnish decreased the size of the lesions and increased the distance of the lesions from the gingival margin when compared to the fluoride varnish, thus decreasing the severity of root caries [139]. Furthermore, a study of the geriatric population found that the chlorhexidine–thymol varnish reduced the size of root caries lesions and decreased the occurrence of new root caries [140]. In an animal model, dental caries were induced in rats by *S. sobrinus* infection [141]. The treatment of gingivae and teeth with a limonene solution was found to reduce the colony-forming units (CFUs) of both total bacteria and *S. sobrinus* compared to the control. Additionally, the number of caries lesions on the smooth surfaces of rat molars was significantly lower in the limonene treatment group. Another in vitro study researched the impact of limonene on ion release, surface microhardness recovery, and hydroxyproline concentration using enamel slabs [142]. Limonene did not express a better action potential than sodium fluoride in preventing demineralization and promoting remineralization.

In dental pulp treatments, hinokitiol can be applied as an endodontic material [143,144]. This monoterpenoid expresses antibacterial properties against *Enterococcus faecalis* [143] and *S. aureus* [144]. Hinokitiol-treated human dental pulp cells had significantly lower COX-2 and interleukin-1 (IL-1) concentrations when compared to the control [143]. Furthermore, odontoblastic differentiation markers (alkaline phosphatase (ALP), dentin matrix protein 1 (DMP-1), and dentin sialophosphoprotein (DSP)) and calcium mineral deposits were positively affected by the presence of hinokitiol. The addition of hinokitiol together with tricalcium silicate-based materials to the calcium phosphate cement (CPC) resulted in reduced working and setting times and increased compressive strength compared to the treatment with CPC alone [144]. There was no improvement in these qualities when CPC was supplemented with hinokitiol only. The addition of hinokitiol significantly increased the radiopacity of the cement samples.

### 3.4. Periodontal Diseases

Severe forms of periodontitis are estimated to affect 19% of the global adult population [130], while less severe gingivitis is thought to be prevalent in 29–88% of the population depending on location, age, and health status [145,146,147]. Periodontitis can result in tooth loss if left untreated. The current treatments involve improving oral hygiene, decreasing bacterial load, and removing calculus by subgingival debridement.

Carvacrol showed various ameliorative effects in rat models [148,149,150]. For example, the use of carvacrol reduced alveolar bone loss in the Wistar rat model compared to the control [148]. The use of carvacrol applied together with chalcones [149], which are naturally occurring polyphenolic compounds that belong to the flavonoids family [151], significantly decreased alveolar bone loss. However, this effect was not observed after treatment with carvacrol alone. Additionally, it was found that both carvacrol alone [148] and carvacrol in combination with chalcones [149] reduced the levels of MPO activity and the number of periodontitis-associated bacteria. When tested in Sprague–Dawley rats with ligation-induced periodontitis, carvacrol treatment decreased alveolar bone loss and the relative mRNA expression of inflammatory indicators such as *Tnfa*, *Il1b*, *Il6*, and *Nos2* [150]. On the other hand, hinokitiol was demonstrated to have anti-inflammatory properties in the BALB/c mouse model [61]. Ligature-induced experimental periodontitis was produced in the animals, and periodontal bone loss was measured in the presence of hinokitiol. The application of hinokitiol resulted in decreased periodontal bone loss and inhibited oral bacterial growth. Furthermore, the relative mRNA transcription in murine gingival tissue of *Il6*, *Il1b*, *Tnf*, and NLR family pyrin domain-containing 3 *(Nlrp3)*, which are associated with pro-inflammatory cytokines, was decreased. Comparable findings were observed within the murine RAW264.7 macrophage cell line, where treatment with hinokitiol decreased the relative mRNA transcription of *Il6*, *Il1b*, and *Tnf*, but not of *Nlrp3* in vitro [61]. A decrease in the levels of pro-inflammatory cytokines would suggest the anti-inflammatory properties of hinokitiol.

In human randomized controlled trial, antimicrobial thymol- and menthol-containing gels were applied as a treatment for gingivitis in orthodontic patients [152]. The patients in the experimental group used the gels between the first (T1) and the second (T2) orthodontic treatment visits and stopped their usage between the second (T2) and the third (T3) visits. The BOP, probing depth, and gingival index (GI) scores decreased in the treatment group while using the gel compared to the placebo control group. Furthermore, a significant difference was observed between the T1–T2 and the T2–T3 time periods for BOP and GI.

Hydrogels can serve as biodegradable scaffolds for periodontal therapy. A thymol-chitosan hydrogel was designed as an alternative periodontal treatment to improve drug delivery and reduce the dosing intervals [153]. An in vitro assessment of the scaffold demonstrated that the application of thymol to the chitosan hydrogel resulted in lower viability and no aggregation of *S. mutans* inside the scaffold when compared to the application of chitosan hydrogel blanks. Microsponges can offer another way of antimicrobial drug delivery during periodontal treatment [154,155]. Eugenyl methacrylate microsponges loaded with eugenol decreased GI and tooth mobility in vivo [154]. Comparable results were observed for thymol microsponges [155].

### 3.5. Mucosal Lesions

Mucosal lesions, commonly referred to as mouth ulcers, can result from various etiologies including oral *Candida*, HSV infections, and cancer. These lesions often cause pain and discomfort and are treated with corticosteroids and antimicrobials [156]. However, due to the side effects associated with these drug classes, less invasive approaches are desired.

To address this, a study was conducted utilizing chemotherapy-induced mucositis in a Wister rat model to determine the efficacy of an oral gel containing borneol [157]. Borneol can be extracted from plants such as wild carrot (*Daucus carota*), common thyme (*T. vulgaris*), common sage (*S. officinalis*), and rosemary (*S. rosmarinus*) and is thought to have anti-inflammatory properties [31,37]. Results demonstrated that in animals treated with a 2.4% borneol oral gel, the cicatrization process improved at the seven-day time point when compared to the control [157]. Additionally, increased collagen levels and decreased inflammatory cell counts were observed. Rats treated with 1.2% and 2.4% borneol lost less weight during a period of 14 days than the control due to improved wound healing. Comparable research was carried out in a Sprague–Dawley rat model using a common guava (*Psidium guajava* L.) extract [158]. Guava flower and leaf extracts are abundant in α-pinene, β-pinene, and limonene. Rats with experimentally induced oral buccal mucosa wounds were exposed to a common guava leaf extract mouthwash, which resulted in a decrease in IL-6 levels in male rats at days 7 and 10, but not at day 14 when compared to the group with induced wounds and no intervention. The same relationship was only observed in female rats on day 14. No significant differences were observed between the control group and the guava treatment groups, except for female rats at day 10. Furthermore, histopathological changes were significantly more severe in the wounded animals’ intervention group when compared to the guava treatment group.

### 3.6. Dry Mouth

A dry mouth can be diagnosed subjectively and objectively; xerostomia is a sensation of having oral dryness, while hyposalivation is characterized by absolute reduced salivation. These symptoms can occur either separately or together, as hyposalivation-induced xerostomia. Treatment involves the use of either products stimulating the salivary flow, or, when the salivary glands have completely lost their function, saliva substitutes such as gels, sprays, or mouthwashes [159,160]. However, these substitutes often have a short-term effect and unpleasant texture and taste, resulting in many patients discontinuing their use [161]. One potential solution is the use of novel therapies based on monoterpenes and their derivatives, which offer a broad selection of flavors and fragrances due to their extensive profiles [162]. Sjögren’s disease patients affected by xerostomia indicated their preference for neutral and menthol-flavored saliva substitutes [163].

Ginger (*Zingiber officinale*) is known for possessing medicinal properties due to its chemical composition rich in terpenes and terpenoids, including monoterpenes and monoterpenoids such as (−)-β-phellandrene, geraniol, geranial (*trans*-citral), eucalyptol, and citral [164,165,166]. To exemplify, C57BL/6 mice were treated with ginger rhizome extracts dissolved in either ethanol or DMSO, and pilocarpine-stimulated salivary flow was measured [167]. The salivary flow rate was found to increase in the intervention groups when compared to their baselines. Furthermore, the ginger rhizome extracts significantly enhanced the salivary flow rate in the intervention groups when compared to the control [167]. In human participants, a study assessed the effectiveness of ginger infusion on salivary secretion in smokers with reduced salivary flow [168]. In this experiment, saliva secretion was determined by sialometry. Ginger infusion was found to increase the unstimulated and stimulated salivary volume after 28 days. In another study, ginger was applied as a saliva stimulant in a form of an oral spray for type II diabetes mellitus [169]. The results using a Schirmer test modified for salivary flow rate revealed an increase in salivary flow after treatment with the ginger spray. The patients also reported a decreased subjective feeling of dry mouth.

Mallow (*Malva sylvestris*) and hollyhock (*Alcea digitata* (Boiss)) are members of the plant family *Malvaceae*. The volatile oil of *M. sylvestris* is rich in monoterpenes and monoterpenoids, with a high percentage of eugenol [170], eucalyptol, α-terpinyl acetate, and menthol [171]. Mallow and hollyhock have both been used in traditional Persian medicine for treating dry mouth [172,173]. A study using the European Organization for Research and Treatment of Cancer Quality of Life Questionnaire, Head and Neck Module (EORTC QLQ-H&N 35), found that head and neck cancer patients reported easier swallowing after using a combination drug of mallow and hollyhock compared to an artificial saliva substitute [173].

Mouthwashes are commonly used in individuals with xerostomia to relieve the feeling of a dry mouth. Licorice (*Glycyrrhizae*) spp. root is abundant in bioactive ingredients, including monoterpenes and their derivatives, such as α-caryophyllene, β-caryophyllene, myrcene, and α-pinene [174]. A study investigated the effects of a licorice mouthwash on salivary flow and xerostomia in hemodialysis patient [175]. It was found that the use of the licorice mouthwash resulted in an increase in unstimulated salivary flow rate compared to the control group. Additionally, the group using the licorice mouthwash reported a decreased feeling of dry mouth compared to both the control group and the group using a water-based mouthwash.

The impact of grapefruit oil, which is abundant in limonene [176], was evaluated on salivary secretion in a Sprague–Dawley rat model [177]. However, there was no significant difference in salivary flow between rats stimulated with grapefruit oil and the control group [177]. In human participants, the benefits of inhaling kuromoji (*Lindera umbellata*) oil and bergamot oil on the salivary function were analyzed [178]. The main volatile components of kuromoji volatile oil are linalool, eucalyptol, and D-limonene [179], while the main constituents of bergamot oil are limonene and linalool [180]. The bergamot volatile oil was found to increase the salivary flow both immediately and 25 min after inhalation, while the kuromoji oil resulted in increased salivation 25 min after inhalation, but not immediately. An organ scanning technology, scintigraphy, was used to measure salivary gland function in individuals following radioactive iodine therapy for thyroid cancer [181]. D-limonene and β-pinene were the primary monoterpene constituents in the applied lemon and ginger (2:1) oil. Aromatherapy with the volatile oil resulted in a significant increase in the accumulation ratio in the parotid and submandibular glands. Furthermore, there was a significant rise in the rate of secretion change before and after the treatment in the bilateral parotid glands. In another study, healthy volunteers were stimulated ortho-nasally with either mastic, which is a resin rich in α-pinene, or pure α-pinene in the form of volatile oil sticks [accepted] [182]. The stimulation with mastic and α-pinene resulted in increased salivary flow when compared to unstimulated participants. Mastic, but not α-pinene, significantly decreased spinnbarkeit, which is a measurement describing the rheology of saliva. These findings suggest that exposure to the mastic odor increased the production of serous saliva. Another human participant study analyzed the impact of a menthol mouth rinse on the salivary flow [183]. Menthol increased the salivary flow and total protein concentration in the treated volunteers compared to the unstimulated participants. Its mode of action is thought to be via an agonistic interaction with the transient receptor potential cation channel subfamily M (melastatin) member 8 channel (TRPM8).

## 4. Side Effects

### 4.1. Allergic Reactions

Monoterpenes are being investigated for their potential beneficial biomedical effects and applications. However, it is important to consider the potential for negative side effects. Eugenol, which used to be commonly employed as a root canal sealer in dentistry, was found to cause hypersensitivity, urticaria, and gingivitis, as well as allergic reactions [184,185,186,187,188,189]. Some reports indicated that eugenol allergy symptoms can resemble burning mouth syndrome [190,191]. Additionally, while uncommon, some toothpaste ingredients [192], including menthol [193] and carvone [194,195,196,197], can cause dermatological issues including contact dermatitis. In line with this, a retrospective study analyzed carvone patch tests results. Of the 4221 individuals tested, 3.48% (N = 147) were positive for carvone sensitization. Within the carvone-positive group, 73% of the participants reported oral signs, and 57% had oral lichens [198].

### 4.2. Toxicity

Monoterpenes and monoterpenoids possess various beneficial properties for the user and generally can be found in professional commodities and over-the-counter products and are used as home remedies. Many of these compounds have been approved for safe application by organs including the European Chemicals Agency (ECHA) [199] and designated as generally recognized as safe (GRAS) [200]. ECHA and the GRAS designation provide guidance and safe concentration ranges for different applications. For example, the maximum acceptable final concentration in products ranges between 0.25 and 7.30% for eugenol [201], 0.017 and 2.30% for citronellal [46], and 0.16 and 7.30% for α-pinene [202]. However, it is important to note that like any other substance, they can become toxic in relatively high concentrations (Table 2).

In a comparative in vitro study examining the impact of selected monoterpenes on human cells, the cell toxicity of camphor, eucalyptol, and thujone was assessed [203]. All three compounds were cytotoxic to MRC-5 fetal lung fibroblasts, with half maximal inhibitory concentration (IC_50_) values of 11 mM for camphor, 11 mM for eucalyptol, and 2.2 mM for thujone. Another form of toxicity, genotoxicity, describes the ability of substances to damage the genetic material in the cell. Camphor, eucalyptol, and thujone showed a genotoxic effect in MRC-5 and Vero cells. However, at concentrations up to 50 μM, the monoterpenes were found to induce a protective response in Vero cells against UV-induced mutagenesis and 4NQO-induced DNA strand breaks. In a comparable study, the results from trypan blue and MTT cytotoxicity assays revealed that citral had cytotoxic and genotoxic effects on peripheral blood mononuclear cells (PBMC) and the human hepatoma cell line HepG2 [205]. Citral was found to be genotoxic to both PBMC and HepG2 cells at a concentration above 50 μg/mL [205], while a camphor derivative, camphorquinone, was found to be genotoxic in the OKF6/TERT-2 telomerase-immortalized cell line above the concentration of 2.5 mM [206].

Various endodontics-focused research articles looked at the composition of root canal sealers. Zinc oxide eugenol root canal sealers were analyzed for their cytotoxicity in human periodontal ligament cells (PDLCs) and a permanent hamster V79 cell line [210]. Eugenol-containing root canal sealers were found to decrease the viability of both PDLCs and V79 cells. Comparable results were reported in human normal oral cells, primary gingival fibroblasts, PDLCs, and hemopoietic progenitor cells [204]. Eugenol was cytotoxic towards these cells with a 50% cytotoxic concentration (CC_50_) above 755 μM.

Injury to the liver tissue can be an outcome of chemical-driven monoterpene toxicity. Some monoterpenes have been proven or are thought to have potential hepatotoxicity in humans in high concentrations, including compounds such as citral, eugenol, camphor, D-limonene, geranial, menthofuran, neral, and pulegone [211,212]. For example, an in vivo analysis found pulegone to be hepatotoxic to rats at relatively high concentrations (400 mg/kg) [207], while limonene was harmful at 25 and 75 mg/kg [208].

Some monoterpenes can also cause neurotoxicity. For example, thujone, which occurs naturally in sage, rosemary, and thyme volatile oils, is used as a common food flavoring and is a constituent of the alcoholic drink absinthe. However, above certain levels, it can have poisonous effects and cause neurotoxicity. A mixture of α- and β-thujone had a median lethal dose 50 (LD_50_) of 192 mg/kg in rats, 230 mg/kg in mice, and 396 mg/kg in guinea pigs [213,214,215]. Another potentially neurotoxic monoterpene, α-terpinene, caused DNA damage, memory deficit, and had overall neurotoxic effects when applied orally in Wistar rats [209].

## 5. Materials and Methods

The review of the literature was performed using the databases of PubMed, Web of Science, Scopus, and Google Scholar until August 2023. The databases were searched for articles using keywords related to (monoterpene), (monoterpenes), (monoterpenoid), (monoterpenoids), (volatile compound), (volatile oil) AND (antimicrobial), (antiseptic), (antibacterial), (antiviral), (antifungal), (anti-cancer), (anti-inflammatory), (analgesic), (anti-nociceptive), (oral hygiene), (oral health), (oral), (mouth), (halitosis), (dentistry), (dental), (dental treatment), (periodontal diseases), (periodontitis), (gingivitis), (mucosal lesions), (dry mouth), (xerostomia), (saliva), (salivary flow), (salivation), (hyposalivation), (side effects), (allergy), (allergic reaction), (toxicity), (toxic). Further manual search was carried out to enroll other potentially relevant articles, which could not be found with the electronic search. Two authors (W.P. and Z.A.) independently searched for articles and examined the title and abstract of all records identified. The authors assessed each of these articles to determine which met the inclusion criteria for this review. For all articles that initially met the inclusion criteria, the full text was read. The inclusion criteria used for the present study were:

- Availability of the full text;
- Appropriate methodology;
- Research regarding pure monoterpenes or monoterpenoids or mixed compounds containing monoterpenes or monoterpenoids.

## 6. Conclusions and Prospects

Monoterpenes and their derivatives are a unique and diverse class of compounds that have only partially been explored. As presented above, they possess significant biological properties, ranging from antimicrobial and anti-inflammatory effects to anticancer activities. However, the true potential of these compounds may lie in their ability to work in combination with modern treatments, potentially enhancing their efficacy and reducing their side effects. Hence, given their potential health benefits for humans, monoterpenes and monoterpenoids represent a promising source for the development of novel compounds that could be applied in general health care, but also specifically in oral health. For example, monoterpenes could be used as natural and effective alternatives to traditional antimicrobial agents. The discovery of molecular targets will lead to a better understanding of the mechanisms of action of the monoterpenes and monoterpenoids of interest and has the potential to support the discovery of new agents showing an improved therapeutic effect and decreased toxic properties. Furthermore, the unique properties of monoterpenes, such as their wide availability, natural origin, and being safe to use, make them attractive candidates for developing new drug (delivery) systems, which could improve the bioavailability and effectiveness of existing therapeutic agents.

However, as monoterpenes have only recently gained attention as potential therapeutic agents in modern health care, there are gaps in the literature that need to be addressed. These include a general lack of knowledge about the optimal dosages, routes of administration, and potential side effects of these compounds. Some of the literature cited in this review suffers from study design flaws, highlighting the need for more rigorous and well-designed studies in the future. New research plans need to be devised to gain a better understanding of how monoterpenes can be utilized in the field of oral health and beyond.

## Figures and Tables

**Table 1:** Biological properties and potential mechanisms of action of selected monoterpenes and monoterpenoids. Chemical structures were obtained from PubChem [18].

Compound Name	Chemical Structure	Occurrence	Biological Properties	Potential Mechanisms
α-Pinene	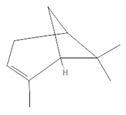	*Cannabis sativa* L. *Curcuma* spp. (turmeric) *Daucus carota* (wild carrot) *Juniperus* spp. (junipers) *Pinophyta* spp. (conifers) *Piper nigrum* (black pepper) [19,20,21,22]	Analgesic and antinociceptive [23]	Interaction with γ-aminobutyric acid type A (GABA_A_) receptor [23]
			Antifungal [24]	Ergosterol complexation [25]
			Antiviral [26]	Interference with the virion envelope or masking of necessary viral structures [26]
α-Terpineol	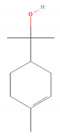	*Citrus aurantium* ssp. amara (bitter orange) *Melaleuca* spp. (tea trees) *Origanium vulgare* L. (oregano) *Pinus* spp. (pines) *Salvia rosmarinus* (rosemary) *Vitex agnus-castus* (chaste tree) *Zingiber officinale* (ginger) [27,28]	Anti-inflammatory [29]	IL-6 inhibition [29]
α-Thujone	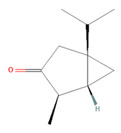	*Artemisia absinthium* (wormwood) *Artemisia herba-alba* (white wormwood) *Artemisia tridentata* (big sagebrush) *Salvia fruticosa* (Greek sage) *Salvia officinalis* (common sage) *Tanacetum vulgare* (tansy) *Thymus* spp. (thymes) [30,31,32]	Anti-inflammatory [31]	Inhibition of PMA/I-induced interleukin-6 (IL-6) and interleukin-8 (IL-8) release [31]
			Antiviral [30]	Unknown
β-Pinene	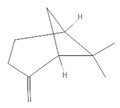	*Anethum graveolens* (dill) *Melaleuca alternifolia* (narrow-leaved paperbark) *Pinus* spp. (pines) *Salvia fruticosa* (Greek sage) *Salvia officinalis* (common sage) *Salvia rosmarinus* (rosemary) *Vitex agnus-castus* (chaste tree) [33]	Anti-cancer [34]	Apoptosis [34]
			Antiviral [35]	Interference with the virion envelope or masking of necessary viral structures [26]
β-Thujone	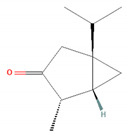	*Artemisia absinthium* (wormwood) *Salvia fruticosa* (Greek sage) *Salvia officinalis* (common sage) *Thuja occidentalis* (northern white cedar) *Thymus* spp. (thymes) [30,31,36]	Anti-inflammatory [31]	Inhibition of PMA/I-induced IL-6 and IL-8 release [31]
			Antiviral [30]	Unknown
Borneol	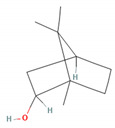	*Daucus carota* (wild carrot) *Mentha spicata* (spearmint) *Salvia officinalis* (common sage) *Salvia rosmarinus* (rosemary) *Thymus vulgaris* (common thyme) *Zingiber* spp. (true gingers) [31,37,38]	Anti-inflammatory [31]	Inhibition of PMA/I-induced IL-6 and IL-8 release [31]
Camphor	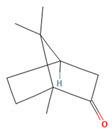	*Artemisia annua* (sweet wormwood) *Cinnamomum camphora* (camphor tree) *Salvia fruticosa* (Greek sage) *Salvia officinalis* (common sage) *Salvia rosmarinus* (rosemary) *Tanacetum vulgare* (tansy) [31,38,39,40]	Anti-inflammatory [31]	Inhibition of PMA/I-induced IL-6 and IL-8 release [31]
			Antiviral [30]	Unknown
Carvacrol	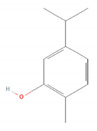	*Lippia origanoides* *Origanum Dictamnus* (dittany of Crete) *Origanum vulgare* (oregano) *Satureja thymbra* (savory of Crete) *Thymus capitatus* (conehead thyme) *Thymus serpyllum* (white thyme) *Thymus vulgaris* (common thyme) [41,42]	Analgesic and antinociceptive [43]	Interaction with opioid, vanilloid, and glutamate systems [43]
Citronellal	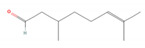	*Corymbia citriodora* (lemon-scented gum) *Cymbopogon* spp. (lemongrass) *Ocimum* spp. (basil) *Zingiber* spp. (gingers) [44,45,46]	Analgesic and antinociceptive [47]	Inhibition of substance P release or neurokinin-1 (NK-1) receptor [47]
Citronellol	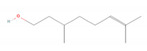	*Citrus* × *deliciosa* (Mediterranean Mandarin) *Corymbia citriodora* (lemon-scented gum) *Cymbopogon* spp. (lemongrass) *Pelargonium* spp. (geraniums) *Rosa* × *damascena* (Damask rose) *Rosa gallica* (Gallic rose) *Zingiber* spp. (gingers) [48,49]	Antifungal [24]	Fungal membrane disruption [50,51]
Eucalyptol (1,8-Cineole)	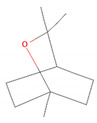	*Curcuma* spp. (turmerics) *Eucalyptus* spp. (eucalyptuses) *Mentha pulegium* (pennyroyal) *Mentha* × *piperita* (peppermint) *Salvia fruticosa* (Greek sage) *Salvia officinalis* (common sage) [30,31,52]	Anti-inflammatory [31]	Inhibition of PMA/I-induced IL-6 and IL-8 release [31]
			Antiviral [26,30]	Interference with the virion envelope or masking of necessary viral structures [26]
Eugenol	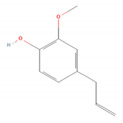	*Cinnamomum* spp. (cinnamon) *Myristica fragrans* (nutmeg) *Ocimum* spp. (basils) *Pimenta dioica* (allspice) *Syzygium aromaticum* (clove) [4,53,54]	Antibacterial [55]	Biofilm disruption [56]
Geraniol	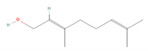	*Camellia sinensis* (tea plant) *Citrus* × *deliciosa* (Mediterranean Mandarin) *Cymbopogon* spp. (lemongrass) *Vitis vinifera* (common grape) *Humulus lupulus* (hops) *Pelargonium* spp. (geraniums) *Rosa* spp. (roses) *Zingiber officinale* (ginger) [49,57,58]	Anti-cancer [59]	Downregulation of nuclear factor kappa B (NF-κB) pathway [59]
Hinokitiol (β-thujaplicin)	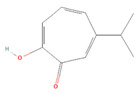	*Chamaecyparis* (false cypresses) *Cupressus* spp. (cypresses) *Thuja* spp. (thujas) *Thujopsis dolabrata* [60]	Anti-inflammatory [61]	Downregulation of mRNA transcription of proinflammatory cytokine-related genes [61]
			Anti-cancer [62]	Induction of apoptosis and autophagy [63]
			Antibacterial [64]	Inhibition of nutrient transport and cell respiration [65]
			Antifungal [66]	Chelating of fungal intracellular iron and respiration inhibition of fungal cells [66]
Isoborneol	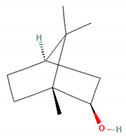	*Artemisia annua* (sweet wormwood) *Curcuma amada* (mango ginger) *Cinnamomum camphora* (camphor tree) *Salvia fruticosa* (Greek sage) *Salvia officinalis* (common sage) *Thymus vulgaris* (common thyme) *Zingiber officinale* (ginger) [67,68]	Antiviral [69]	Inhibition of viral glycosylation [69]
Limonene	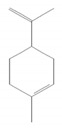	*Anethum graveolens* (dill) *Citrus* spp. (citruses) *Eucalyptus globulus* (southern blue gum) *Melaleuca alternifolia* (narrow-leaved paperbark) *Salvia officinalis* (common sage) *Salvia rosmarinus* (rosemary) *Vitex agnus-castus* (chaste tree) [70,71]	Antifungal [24]	Disruption of cell wall and cell membrane, which leads to apoptosis [72]; inhibition of adhesion, enzyme secretion, and biofilm formation [73]
			Antiviral [35,74]	Interference with virion envelope structures or masking of necessary viral structures [35]
Menthol	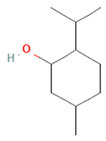	*Mentha* spp. (mints) [75]	Antiviral [76]	Attenuation of infection by stimulating TRPM8, which blocks TRPV1-mediated mitochondrial fragmentation [76]
Myrtenol	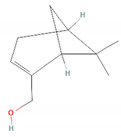	*Achillea* spp. (yarrows) *Eucalyptus* spp. (eucalyptuses) *Myrtus communis* (common myrtle) *Taxus* spp. (yews) [77,78]	Analgesic and antinociceptive [79]	Cytokine inhibition and p38 mitogen-activated protein kinase (p38-MAPK) signal transduction pathway in trigeminal ganglia [79]
Perillic acid	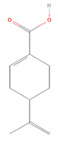	*Perilla frutescens* (beefsteak plant) [80,81]	Antiviral [82]	Inhibition of maturation, fusion, and viral infectivity [82]
Perillyl alcohol	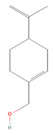	*Carum carvi* (caraway) *Lavandula × intermedia* (lavandin) *Mentha piperita* (peppermint) *Mentha spicata* (spearmint) *Perilla frutescens* (beefsteak plant) [83,84,85]	Antiviral [82]	Inhibition of maturation, fusion, and viral infectivity [82]
Sabinene	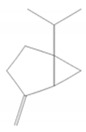	*Artemisia annua* (sweet wormwood) *Juniperus communis* (common juniper) *Mesosphaerum suaveolens* (pignut) *Piper nigrum* (black pepper) *Salvia rosmarinus* (rosemary) *Vitex agnus-castus* (chaste tree) *Zingiber montanum* [86,87,88]	Antibacterial [86,89]	Inhibition of bacterial adherence, growth and biofilm formation [89]
Thymol	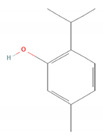	*Lippia* spp. *Nigella sativa* (black caraway) *Ocimum* spp. (basils) *Origanum* spp. (oreganos) *Satureja thymbra* (savory of Crete) *Thymus* spp. (thymes) *Trachyspermum ammi* (ajowan caraway) [90,91]	Antibacterial [92]	Biofilm disruption, decrease in virulence [92,93], reduction of viability and metabolic activity, autolysis [93]
			Anti-cancer [94]	Mitochondria-mediated apoptosis [94]
			Antifungal [95]	Disruption of cell membrane integrity [95]

**Table 2:** Overview of the toxicity of selected monoterpenes and their derivatives. IC_50_—half maximal inhibitory concentration, CC_50_—50% cytotoxic concentration, LD_50_—median lethal dose 50.

Type of Toxicity	Compound Name	Type of Assay	Cell Line/Animal	Toxicity
Cytotoxicity	Camphor	In vitro	MRC-5 fetal lung fibroblasts	IC_50_ 11 mM [203]
	Eucalyptol	In vitro	MRC-5 fetal lung fibroblasts	IC_50_ 11 mM [203]
	Thujone	In vitro	MRC-5 fetal lung fibroblasts	IC_50_ 2.2 mM [203]
	Eugenol	In vitro	Primary gingival fibroblasts, hemopoietic progenitor cells, human periodontal ligament cells	CC_50_ > 755 μM [204]
	Citral	In vitro	Peripheral blood mononuclear cells, human hepatoma cell line HepG2	≥50 μg/mL [205]
Genotoxicity	Camphorquinone	In vitro	OKF6/TERT-2 telomerase-immortalized cells	≥2.5 mM [206]
	Pulegone	In vivo	Male albino rats (Indian Institute of Science strain)	400 mg/kg [207]
Hepatotoxicity	Limonene	In vivo	Male Wistar rats	25 mg/kg, 75 mg/kg [208]
Neurotoxicity	Thujone	In vivo	Rats, Mice, Guinea Pigs	LD_50_ 192 mg/kg (rats), 230 mg/kg (mice), 396 mg/kg guinea pigs [205]
	α-Terpinene	In vivo	Female Wistar rats	≥0.5 mL/kg [209]

## Data Availability

Not applicable.
